# Reframing Non-Communicable Diseases and Injuries for Equity in the Era of Universal Health Coverage: Findings and Recommendations from the Kenya NCDI Poverty Commission

**DOI:** 10.5334/aogh.3085

**Published:** 2021-01-05

**Authors:** Kibachio Mwangi, Gladwell Gathecha, Mary Nyamongo, Sylvester Kimaiyo, Jemima Kamano, Fredrick Bukachi, Frank Odhiambo, Hellen Meme, Hussein Abubakar, Nelson Mwangi, Joyce Nato, Samuel Oti, Catherine Kyobutungi, Marylene Wamukoya, Shukri F. Mohamed, Emma Wanyonyi, Zipporah Ali, Loise Nyanjau, Ann Nganga, Dorcas Kiptui, Alfred Karagu, Mary Nyangasi, Valerian Mwenda, Martin Mwangi, Aaron Mulaki, Daniel Mwai, Paul Waweru, Mamka Anyona, Peninah masibo, David Beran, Idris Guessous, Matt Coates, Gene Bukhman, Neil Gupta

**Affiliations:** 1 Division of Non-Communicable Diseases, Ministry of Health, Nairobi, Kenya; 2 Institute of Global Health, Faculty of Medicine, University of Geneva, Geneva, Switzerland; 3 World Health Organization (WHO) South Africa Country Office, South Africa; 4 African Institute of Health and Development, Nairobi, Kenya; 5 Academic Model Providing Access to Healthcare, Eldoret, Kenya; 6 Department of Clinical Medicine and Therapeutics, University of Nairobi, Nairobi, Kenya; 7 Kenya Medical Research Institute, Nairobi, Kenya; 8 Field Epidemiology and Laboratory Training Program, Nairobi, Kenya; 9 World Health Organization Country Office, Nairobi, Kenya; 10 International Development Research Center, Nairobi, Kenya; 11 African Population and Health Research Center, Nairobi, Kenya; 12 International Institute For Legislative Affairs, Nairobi, Kenya; 13 Kenya Hospices and Palliative Care Association, Nairobi, Kenya; 14 RTI International, Nairobi, Kenya; 15 School of Economics, University of Nairobi, Nairobi, Kenya; 16 Kenya National Bureau of Statistics, Nairobi, Kenya; 17 Program in Global Non-Communicable Diseases and Social Change, Department of Global Health and Social Medicine, Harvard Medical School, Boston, USA; 18 Global Programs for Research and Training affiliate of The University of California San Francisco, USA; 19 Moi University School of Public health, Eldoret, Kenya; 20 Division of Primary Care Medicine, Geneva University Hospital and University of Geneva, Geneva, Switzerland; 21 Division of Tropical and Humanitarian Medicine, University of Geneva and Geneva University Hospitals, Geneva, Switzerland; 22 NCD Synergies, Partners In Health, Boston, USA

## Abstract

**Background:**

Kenya has implemented a robust response to non-communicable diseases and injuries (NCDIs); however, key gaps in health services for NCDIs still exist in the attainment of Universal Health Coverage (UHC). The Kenya Non-Communicable Diseases and Injury (NCDI) Poverty Commission was established to estimate the burden of NCDIs, determine the availability and coverage of health services, prioritize an expanded set of NCDI conditions, and propose cost-effective and equity-promoting interventions to avert the health and economic consequences of NCDIs in Kenya.

**Methods:**

Burden of NCDIs in Kenya was determined using desk review of published literature, estimates from the Global Burden of Disease Study, and secondary analysis of local health surveillance data. Secondary analysis of nationally representative surveys was conducted to estimate current availability and coverage of services by socioeconomic status. The Commission then conducted a structured priority setting process to determine priority NCDI conditions and health sector interventions based on published evidence.

**Findings:**

There is a large and diverse burden of NCDIs in Kenya, with the majority of disability-adjusted life-years occurring before age of 40. The poorest wealth quintiles experience a substantially higher deaths rate from NCDIs, lower coverage of diagnosis and treatment for NCDIs, and lower availability of NCDI-related health services. The Commission prioritized 14 NCDIs and selected 34 accompanying interventions for recommendation to achieve UHC. These interventions were estimated to cost $11.76 USD per capita annually, which represents 15% of current total health expenditure. This investment could potentially avert 9,322 premature deaths per year by 2030.

**Conclusions and Recommendations:**

An expanded set of priority NCDI conditions and health sector interventions are required in Kenya to achieve UHC, particularly for disadvantaged socioeconomic groups. We provided recommendations for integration of services within existing health services platforms and financing mechanisms and coordination of whole-of-government approaches for the prevention and treatment of NCDIs.

## INTRODUCTION

The focus of the prevention and control of non-communicable diseases (NCDs) has been on four major diseases and four risk factors, as framed by the World Health Organization (WHO) in the Global Action Plan (GAP) for the Prevention and Control of Non-communicable Diseases in 2013 [1]. The emphasis on tobacco control, alcohol control, physical activity, and healthy diet as well as cardiovascular diseases, cancer, chronic obstructive pulmonary disease, and cancers was put forward as a global agenda to consolidate focus on the complex interplay between the socio-economic, environmental and modifiable behavioral factors that underlie the causation and distribution of these chronic conditions.

While this four by four framework has provided valuable guidance to strategic planning and activities for the prevention of control of non-communicable diseases and injuries (NCDIs) in countries such as Kenya, where these conditions form a considerable share of national morbidity and mortality [2], it was recently expanded during the 2018 UN high level meeting to a five by five that now includes air pollution and mental health.

However, recent studies have suggested that a large proportion of the global DALYs due to NCDIs may be due to risk factors and conditions other than those represented in this framework [3]. This differential burden of NCDs and risk factors may be particularly pronounced in younger populations and those living in extreme poverty, as is present in a large proportion of the Kenyan population. Although data from primary studies is limited, a higher prevalence has been shown in urban and wealthier demographic groups in Kenya for several common and lifestyle-associated NCDIs, such as hypertension [4], diabetes [5,6,7], and chronic respiratory diseases [8,9], as compared to more rural populations and lower socioeconomic groups. Meanwhile, the prevalence may be higher for several severe and highly disabling NCDI conditions, such as esophageal cancer [10], epilepsy [11], mental disorders [12], suicide [13], violent injuries [14,15], intimate partner violence [16,17], falls [18], animal bites [18], and burns [18,19].

The economic impact of NCDIs in Kenya is more impoverishing than communicable diseases and is more pronounced in the poor [20]. For households reporting NCDs in a nationally-representative household survey, 29.9% of those in the lowest quintile experienced catastrophic expenditures (defined as >30% of total household income), compared to 9.2% in the highest income quintile [21]. The rate of catastrophic expenditure was also higher for rural (20.8%) as compared to urban (13.6%) households.

It is in this context that the Kenya NCDI Poverty Commission (herein “Commission”) was established and launched by the Kenya Ministry of Health in December 2017, in collaboration with the Lancet Commission on Reframing Non-Communicable Diseases and Injuries for the Poorest Billion. The Commission was comprised of representatives from the Ministry of Health (MOH), academic institutions, WHO, World Bank, research institutions, civil society, not-for-profit organizations, and other key cross-sectoral stakeholders in NCD control. The intent of this Commission was to use existing data sources to best summarize the impact of NCDIs on the health of Kenyans, establish the relationship of poverty with NCDIs in Kenya, develop a proposed package of health sector interventions to raise the visibility and understanding of this problem among policy makers and civil society in Kenya, and inform future planning and resource allocation. We here present the key findings and recommendations from the Kenya NCDI Poverty Commission (full report available at: *http://www.ncdipoverty.org/kenya-report*) [22].

## METHODS

A literature review on NCDIs was conducted, consisting of all studies published from 2006–2016 (extended to January 31st, 2017). The search terms corresponded to the Global Burden of Disease (GBD) “level 2 NCDI” categories combined with the word “Kenya” [23]. Studies were included if they met any of the following criteria: (1) contained data on prevalence, risk or mortality from NCDIs preferably stratified by socioeconomic strata or by geographic location; (2) reported distributions of types of NCDI cases among admissions and deaths at health facilities; and (3) reported on interventions or service delivery models for NCDIs.

The GBD 2016 study was utilized to model and estimate prevalence, disability-adjusted life years (DALYs), and percent of total deaths for specific NCDs, injuries, and risk factors [23]. The burden distribution was further analyzed by age and year. Data from the Kenya Demographic and Health Survey (DHS) were used to gather nationally-representative household surveys that provide data for a wide range of monitoring and impact evaluation indicators in the areas of population, health, and nutrition [18]. This survey also contained data on tobacco use, alcohol use, and adult nutritional status among men and women aged 15–49 years disaggregated by wealth quintile. Data from the Kenya Medical Research Institute (KEMRI)/Centers for Disease Control and Prevention (CDC) Health and Demographic Surveillance System (HDSS) in rural Siaya County in western Kenya and the Nairobi Urban HDSS in two urban slum communities in Nairobi were analyzed to determine cause of death and overall death rates due to NCDIs and calculate comparative distribution of NCDIs by socioeconomic status [15,24].

The population in each county living with at least four levels deprivations from an adapted Multidimensional Poverty Index (MPI) were used to construct an index of socioeconomic indicators from the Kenya DHS, including schooling, school attendance, electricity, sanitation, water, flooring, cooking fuel, and household assets [25].

Baseline availability of services was estimated using the 2013 Kenya Service Availability and Readiness Assessment (SARA) [26]. Availability of services was analyzed by disease condition, level of the health system, rural or urban location, and county. Both reported availability of services and readiness by the observed availability of designated tracer items were analyzed. The association of the percentage of health facilities with service/medication availability or readiness with proportion of households living in poverty per the MPI across counties was tested using Pearson’s correlation coefficient and the p-value of the slope from a simple least squares regression. Essential medications for NCDIs were obtained from the Kenya Essential Medications List 2016 [27]. The Kenya STEP Survey 2015 was reviewed to assess behavioral and metabolic risk factors for NCDs [14]. Data from this survey was secondarily analyzed to obtain wealth quintile disaggregation for survey questions pertaining to diagnosis and treatment for hypertension and diabetes. Availability of referral level services was provided by experts’ report from the members of the Commission.

Data regarding national health expenditures were extracted from the Kenya National Health Accounts 2015/2016 [28]. These data were reviewed and validated by commissioners to establish baseline availability and financing of services.

## KEY FINDINGS

### OVERALL BURDEN OF NCDIS IN KENYA

**Figure 1:**
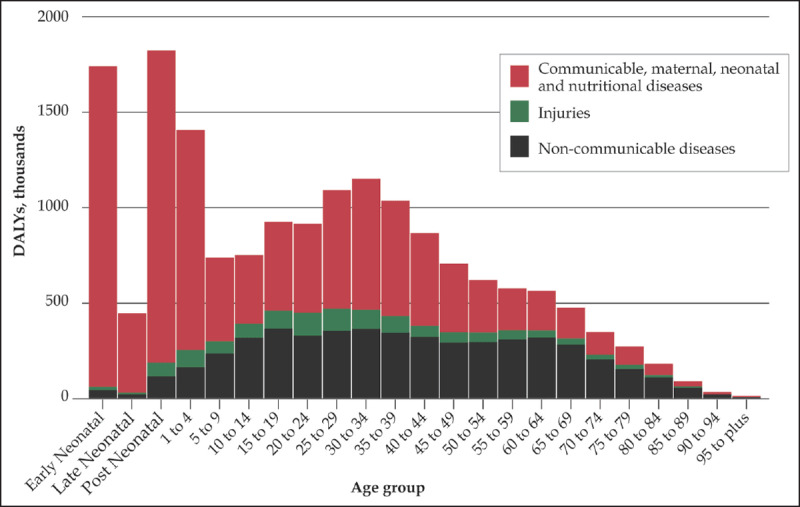
Disability-adjusted life years due to major disease groups, by age (GBD 2016).

**Figure 2:**
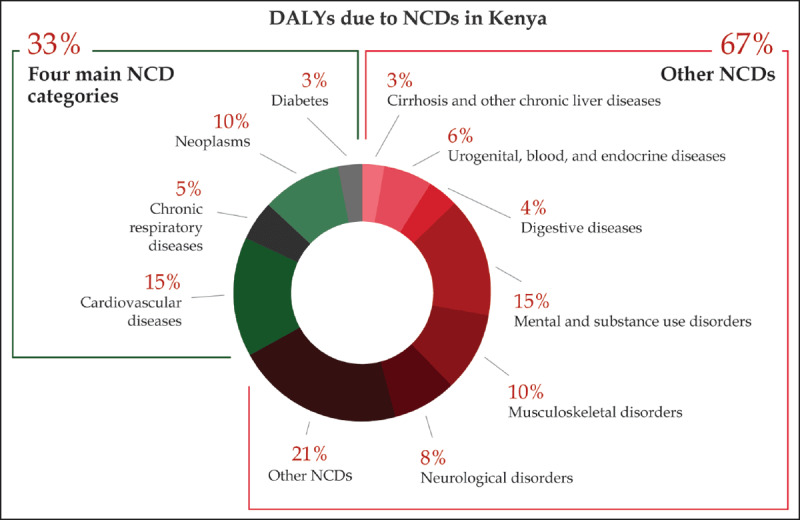
DALYs from NCDs in Kenya due to the four NCDs included and not included in the Global Action Plan (GBD 2016).

Overall, NCDIs accounted for 37% of the disease burden in DALYs in Kenya with more than half (53%) of NCD DALYs and 72% of injury DALYs occurring before the age of 40 (Figure 1). Of all DALYs from NCDs, 67% were related to conditions other the four NCDs highlighted in the WHO GAP (Figure 2). This is notably higher than in high-income countries, where only 53% of NCD DALYs are due to conditions other than these four disease areas [23]. NCD disease categories with the highest proportion of DALYs occurring under the age of 40 included mental health disorders (74%), neurological disorders (70%), and chronic respiratory diseases (50%). NCDIs were responsible for 35% of all deaths in Kenya, and 22% of these occurred before the age of 40.

### BURDEN OF NCDIS IN RELATION TO SOCIOECONOMIC STATUS

Data from the KEMRI/CDC HDSS in Siaya County, a primarily rural catchment area located in the western region of the country from 2003–2016, reported 36.1% of deaths were attributed to NCDs and 4.6% were attributed to injuries by verbal autopsy methods. Individuals in the lowest wealth quintile had a comparable proportion of deaths due to NCDIs (39.0%) as compared to the highest wealth quintile (41.6%) (Figure 3a). The leading causes of NCD deaths among the poorest quintile were cancers (32.1%), cardiovascular disease (26.3%), and abdominal causes (16.2%). Using the multidimensional poverty index to assess poverty, 39.5% of Kenyans would be considered as living in the poorest billion people globally.

**Figure 3a:**
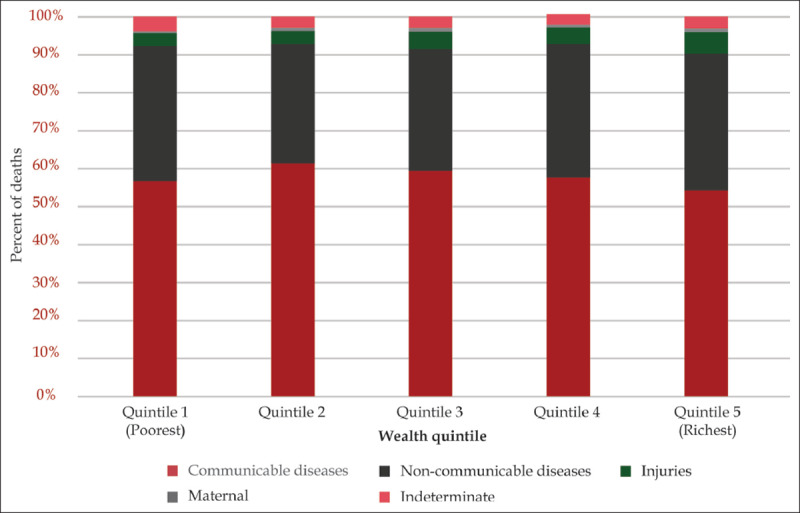
Proportion of cause of death by wealth quintile in KEMRI/CDC HDSS in Siaya County 2003–2016 (source: KEMRI/CDC HDSS 2017).

Data from the Nairobi Urban HDSS from 2010–2015 demonstrated overall a lower proportion of deaths due to NCDs (14%) compared to the rural based Siaya HDSS but a much higher proportion of deaths due to injuries (19%). The proportion of deaths due to NCDs was relatively constant across wealth quintiles (range: 12.1–15.2%), but the proportion of deaths due to injuries was highest in the poorest quintile (22.8%) as compared to the wealthiest quintile (16.9%). Overall, crude death rates due to both NCDs and injuries demonstrated a clear socioeconomic trend, with increasing death rates associated with increasing level of poverty (Figure 3b).

**Figure 3b:**
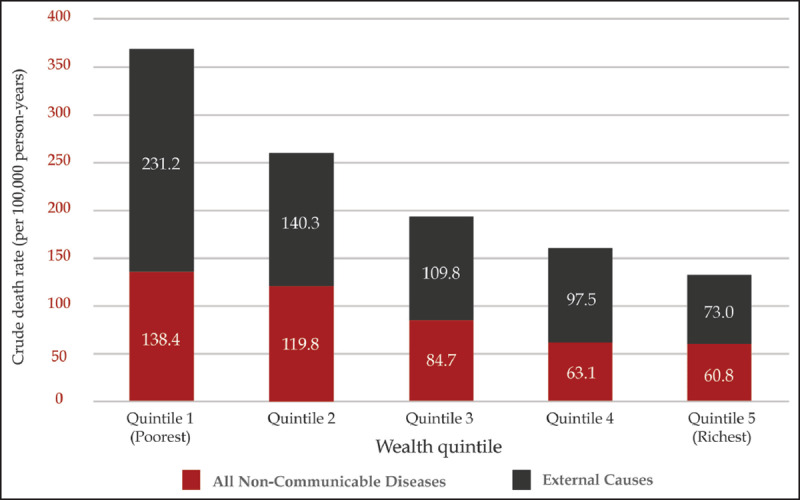
Crude death rates due to NCDs and injuries by wealth quintile in Nairobi Urban HDSS, 2010–2015 (Source: APHRC 2017).

### RISK FACTORS FOR NCDIS IN KENYA

**Figure 4:**
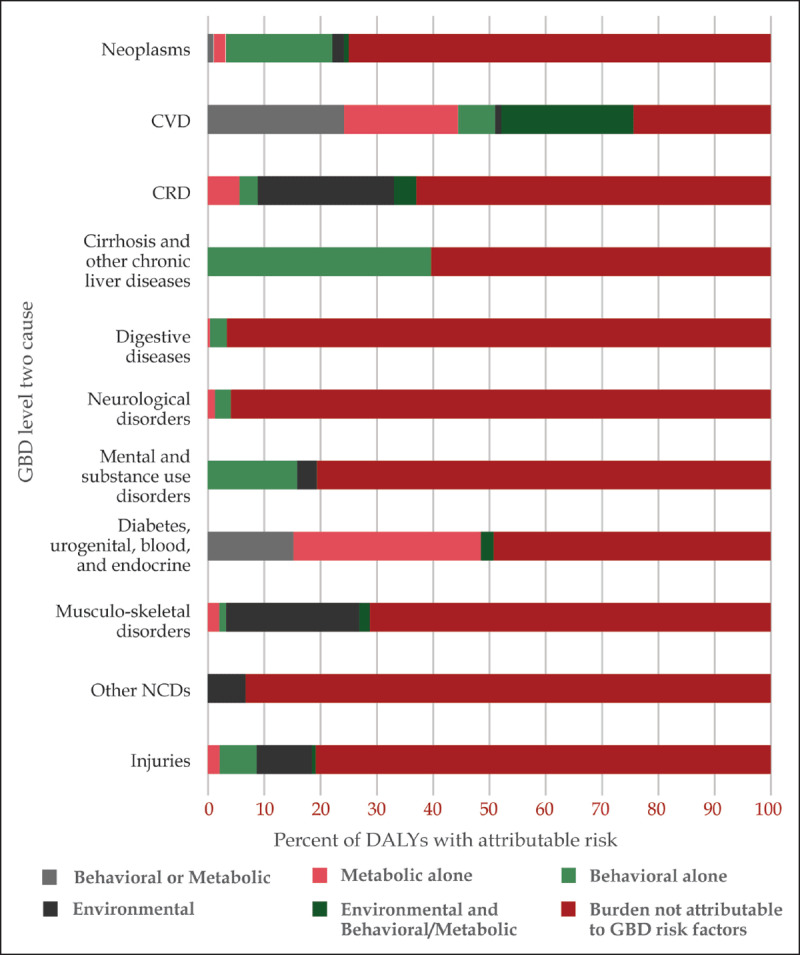
Proportion of disability-adjusted life years in Kenya due to risk factor groups in each NCDI disease category (GBD 2016).

In risk factor modeling from GBD 2016, behavioral and metabolic risk factors, such as tobacco, alcohol, obesity and raised blood pressure, accounted for only 21% of DALYs from NCDI conditions in Kenya. In this model, 73% of all NCDI DALYs were not attributable to the examined risk factors. In regard to DALYs associated with NCDs alone, 23% of these DALYs were attributed to behavioral and metabolic risk factors, while the four traditional behavioral risk factors were associated with 13% of all NCD DALYs. In the categories of digestive diseases, neurologic diseases, musculoskeletal diseases, and other NCDs, virtually none of the risk factor profile could be attributed to behavioral or metabolic causes (Figure 4). For injuries and mental and substance use disorders, only 10–15% of the risk factor profile was attributed to behavioral causes.

Although the categories of neoplasms and cirrhosis had a higher proportion of attributable risks to behavioral risk factors (20% and 53%, respectively), which likely represents the relationship of smoking with lung cancer and alcohol use with cirrhosis, it is notable that the vast majority of neoplasms and almost half of cases of cirrhosis were caused by other risk factors, such as chronic infections (human papilloma virus, Epstein Barr virus, hepatitis B and C) or genetic predispositions. Chronic respiratory diseases had the largest component of risk factors attributable to the environment, presumably through air pollution and indoor pollution due to cooking using solid fuels (such as wood, crop wastes, charcoal, coal, and dung) and kerosene in open fires and inefficient stoves. Although the categories of both cardiovascular disease and diabetes had the highest proportion of metabolic risk, metabolic risk alone remained less than half of the risk factor profile for each of these categories, which suggests a large component of non-metabolic related disease conditions, such as rheumatic heart disease, cardiomyopathies, and type 1 diabetes, within these categories.

### SERVICE AVAILABILITY FOR NCDIS IN KENYA

**Figure 5:**
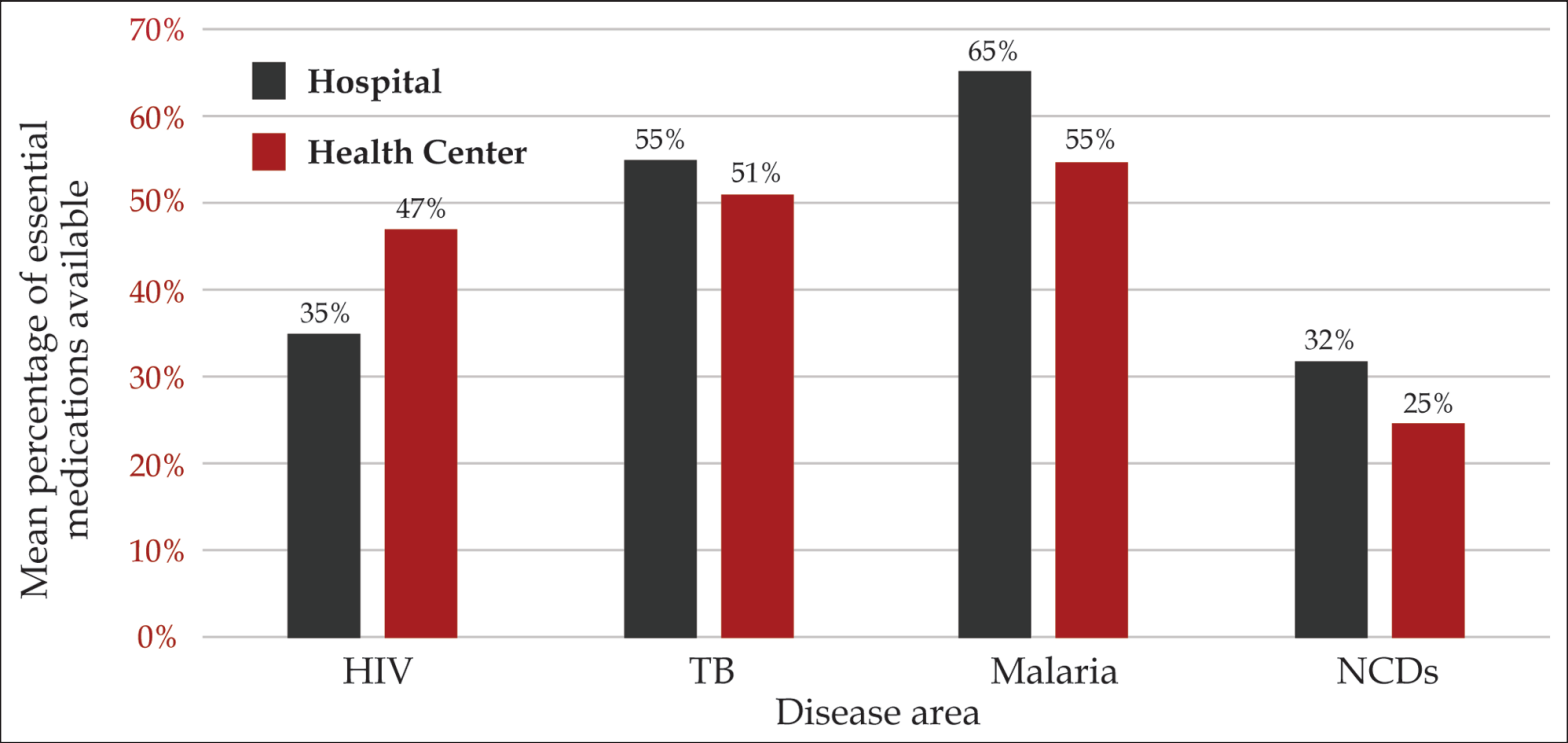
Mean availability of essential medications for NCDs compared to HIV, TB, and malaria (Service Availability and Readiness Assessment, 2013).

Overall, 34% of facilities surveyed in the SARA 2013 health facility survey were considered ready for NCD services as measured by the presence of standard precautions, basic amenities, basic equipment, and essential medicines. The level of readiness was 51% of hospitals, 51% at health centers, 36% at dispensaries, and 21% at medical clinics. A greater proportion of public facilities (42%) were considered ready as compared to private not-for-profit (34%) and private for-profit (22%) facilities. Overall, all facilities had available 37.1% of components of this readiness package, and only 4.9% of facilities had all components available. Compared to other disease areas, essential NCD medications were less available at both hospitals (32%) and primary care facilities (25%) than medications for malaria (65% and 55%, respectively), tuberculosis (TB) (55% and 51%, respectively), and HIV (35% and 47%, respectively) (Figure 5).

Among medications on the national essential medicines list, there was low availability of medicines in both urban (31%) and rural health facilities (22%) (Figure 6a). Only glucose injectable, furosemide, and paracetamol were more available in the public health facilities. Overall, counties with a higher proportion of the population living in the poorest billion had a lower mean number of tracer NCD medications available as compared to counties with a lower proportion of population living in the poorest billion (r = –0.386, p = <0.01) (Figure 6b). Only the availability of aspirin and insulin were not correlated with poverty level in the counties.

**Figure 6a:**
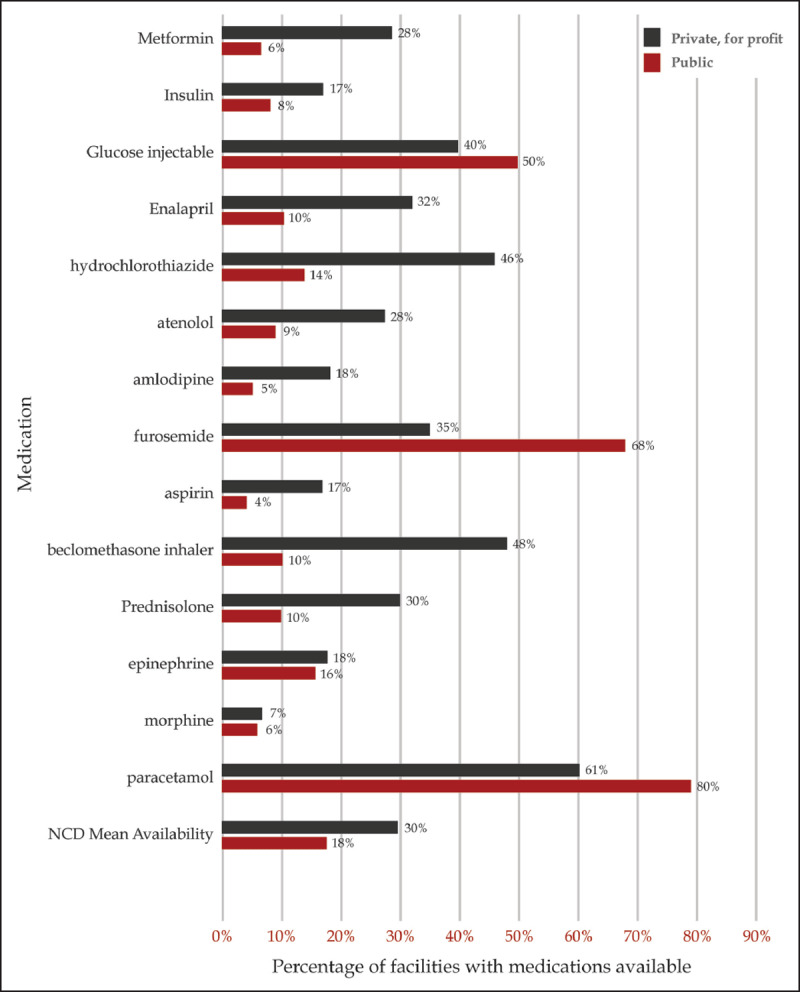
NCD medication availability in all facilities by urban or rural categorization (Service Availability and Readiness Assessment, 2013).

**Figure 6b:**
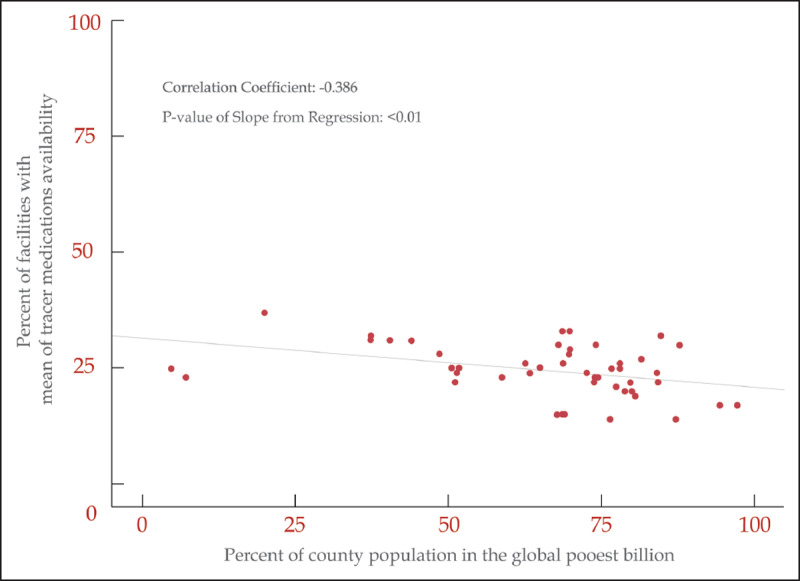
Percent of facilities in each county with the mean number of tracer medications available for NCDs by the percent of the county’s population living in the global poorest billion (Sources: Service Availability and Readiness Assessment 2013 and Oxford Poverty & Human Development Initiative).

The distribution of NCDI referral services listed in Kenya Essential Package for Health (KEPH) and specialty physicians throughout the country has not been well quantified, though such resources are limited and largely concentrated in urban areas. Expert opinion provided by the Commission indicates that currently there were a limited number of referral level services in the public sector in Kenya. These include six cardiac surgery centers, twelve centers with capacity for chemotherapy, and six radiotherapy centers, though only one radiotherapy center in the public sector. Through the “Changing Diabetes in Children” project, there are eight “hubs” providing care for type 1 diabetes in the public sector, with 16 “spokes”. Dialysis is more readily available, with approximately 294 centers around Kenya. Computed tomography (CT) is available at 17 public facilities, and magnetic resonance imaging (MRI) at 20 public facilities. Histopathology services are available at most major hospitals, and 11 county hospitals have integrated palliative care services and serve as training and mentorship sites for county hospitals [29,30]. Availability of these services as well as specialist physicians per 100,000 population were calculated by the Commission and full results are available in the Kenya NCDI Poverty Report [22].

### ACCESS AND COVERAGE OF BASIC NCDI SERVICES

**Figure 7:**
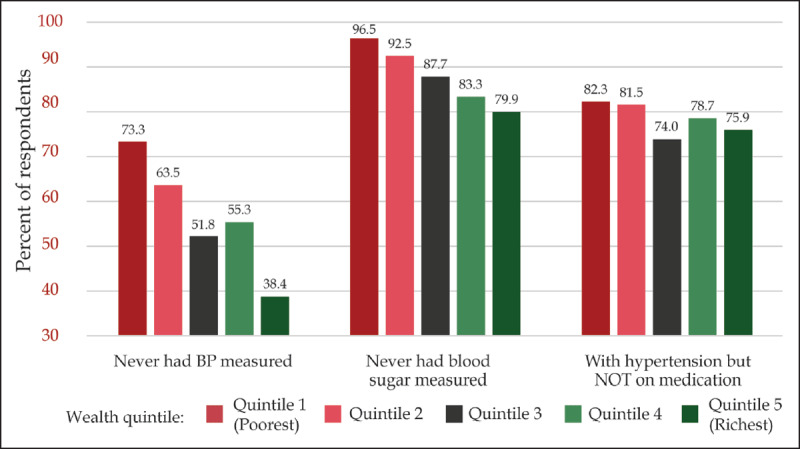
Access to diagnosis for hypertension and diabetes and treatment for hypertension, by wealth quintile (STEPS 2013).

According to the Kenya STEP survey 2015, 55.8% of respondents had never had their blood pressure measured in the past, and 87.8% had never had their blood sugar measured in the past [14]. Access to screening for both hypertension and diabetes was related to wealth quintile, with progressively higher proportions never previously screened with increasing poverty level (Figure 7). This relationship was also seen when comparing urban and rural populations, with a higher proportion of individuals never previously screened in rural areas for both hypertension (60.7% vs. 48.1%) and diabetes (89.6% vs. 84.8%). Of those patients found to have hypertension, access to treatment was associated with wealth quintile, with poorer populations less likely to be on treatment (Figure 7). A higher proportion of patients were screened and on treatment for hypertension from urban areas (24.8%) than from rural areas (20.3%) [14].

In regard to coverage of other NCDI services, the distribution of cancer screening suggested a socioeconomic trend. In the Kenya DHS 2014, cancer screening was higher among those in the wealthiest quintile as compared to those in the poorest quintile for breast cancer (22.7% vs. 4.4%, respectively), cervical cancer (22.9% vs. 4.4%), and prostate cancer (3.9% vs. 1.5%) [18]. Screening for each of the above cancers was also found to be higher in urban compared to the rural areas.

### CURRENT SPENDING ON NCDIS IN KENYA

According to the Kenya National Health Account (NHA) Fiscal Year (FY) 2015/16, total health expenditure (THE) in Kenya was $3,475,539,658 USD or $78.60 USD per capita. This THE was 5.2% of nominal gross domestic product, and the government expenditure on health was 6.7% of all government expenditure. Expenditure on NCDs in FY 2015/16 was $198,568,740 USD, or 5.7% of THE ($4.48 USD per capita), a decrease from 6.2% in FY 2012/13. Of this expenditure on NCDs, 45% of revenue for financing schemes was from the government, 31% from employers, 20% from households, and 5% from international sources. Two-fifths (40%) of the expenditures for NCDs were incurred in government health facilities while 34% was incurred in private health facilities. About half (48%) of NCD expenditures were for outpatient curative care services, 29% for inpatient curative care, and 9% for preventive care. Expenditure on injuries in 2015/16 was an additional 3.7% of THE ($2.91 USD per capita), a decrease from 4.1% in FY 2012/13 [28].

## PRIORITY NCDI INTERVENTIONS FOR UNIVERSAL HEALTH COVERAGE

The Commission undertook a process to prioritize among NCDIs conditions for the Kenya health sector based on principles of priority setting established by the WHO Consultative Group on Equity and Universal Health Coverage (UHC) [31]. The Commission analyzed and ranked NCDI conditions based on the estimated burden of disease of each condition in Kenya as measured by DALYs in the GBD 2016 [23]. The severity of each condition was measured using the average years of life lost (YLL) per death, and the disability of each condition was measured using the years of life with disability (YLDs) per incident case. The impact in Kenya compared to high-income countries was estimated for each condition by comparing the rate of DALYs per 100,000 population. A total of 190 NCDI conditions from the GBD database were analyzed by these metrics, and a summary score was provided to each condition according to the average of the ranking quartiles on each metric. The 50 conditions with the highest summary score were then reviewed by a sub-committee of the Commission. The commissioners then selected a final set of 14 conditions based on: 1) their ability to contribute significantly to adverse health and economic consequences in Kenya, 2) how feasible and effectively they can be controlled in Kenya, and 3) their being complementary to ongoing strategy and efforts by the Government of Kenya (GoK) as per the national health policy and NCD strategic plan. The selected conditions are shown in Table 1.

**Table 1:** Selected priority NCDIs for service expansion.


DISEASE CATEGORY	PRIORITIZED DISEASE/CONDITION

Respiratory	Asthma

	Chronic obstructive pulmonary disease

Cardiovascular – behavioral & metabolic etiologies	Hypertension, Hypertensive heart disease, Ischemic heart disease, hemorrhagic stroke, ischemic stroke

Cardiovascular – other etiologies	Rheumatic heart disease

Endocrine	Diabetes mellitus (type 1 and 2)

Cancers	Cervical cancer

	Burkitt lymphoma (non-Hodgkin lymphoma)

	Breast cancer

Mental Health	Major depressive disorder

Neurologic	Epilepsy

Congenital	Sickle cell disorders

Liver	“Cirrhosis” – etiologies include hepatitis B, hepatitis C, alcohol, other causes

Surgical & Injuries	Motor vehicle road injuries

	Assault


Information regarding evidence-based and cost-effective health sector interventions for NCDIs was obtained from Disease Control Priorities 3 (DCP3) [32]. The unit cost for each intervention was provided on average across low-income countries, which is described in greater detail elsewhere [33]. Tradable costs were adjusted for the cost of health sector expenditure in Kenya, and an estimated 50% indirect cost was added to the total cost for each intervention. These interventions were reviewed by the Commission for 1) alignment with stated NCDI priority conditions, and 2) feasibility and desirability in the Kenya context. Each intervention was assigned a baseline coverage in Kenya, estimated from existing data sources as well as expert experience from the commissioners. With consultation from national and regional health managers, the Commission then assigned a feasible target coverage for each intervention by the year 2030. The total cost of implementing these interventions and premature deaths averted was then calculated [34].

The total annual cost of the incremental increase in coverage represented by this package is estimated to be $520,146,154 USD, or approximately KSh 54.7 billion (Table 2). This package of interventions would represent a 2.6-fold increase in the current expenditure on NCD services as reported by the NHA 2015/16, which would comprise 15.0% of the current total health expenditure. Overall, this package of interventions, which includes outpatient, inpatient, surgical, mental health, rehabilitation, and palliative care services would represent an incremental investment of $11.76 per capita annually. Using mortality impact estimation methodology described by the DCP3 group, these interventions, if implemented to target coverage, are projected to avert 9,322 premature deaths per year by the year 2030. This figure represents an approximate 10% reduction in expected premature deaths by the year 2030 (according to 2015 death rates).

**Table 2:** Selected priority interventions for NCDIs with cost-effectiveness rating, financial risk protection rating, equity rating, total cost for estimated population in need, estimated baseline coverage, assigned target coverage, and calculated incremental cost.


CONDITION	INTERVENTION	COST EFFECTIVENESS RATING	FINANCIAL RISK PROTECTION RATING	EQUITY RATING	TOTAL COST	BASELINE COVERAGE 2018	TARGET COVERAGE 2030	INCREMENTAL COST	HEALTH SYSTEM LEVEL

Cirrhosis	Screening and brief intervention for alcohol use disorder	3	2	1					

Respiratory	Low-dose inhaled corticosteroids and broncho-dilators for asthma and for selected patients with COPD	1	3	1	262,690,039	0.16	0.5	89,314,613	Health Center

Respiratory	Management of acute exacerbations of asthma and COPD using systemic steroids, inhaled beta-agonists, and, if indicated, oral antibiotics and oxygen therapy	1	4	1	165,654,576	0.16	0.5	56,322,556	First-Level Hospital

Respiratory	Mass media for awareness on handwashing and household air pollution health effects	0	1	1	1,768,986	0.1	0.8	1,238,290	Population

Respiratory	Tobacco cessation counseling and use of nicotine replacement therapy in certain circumstances	4	2	1	46,322,061	0.07	0.6	24,550,692	Health Center

Respiratory	Mass media messages concerning use of tobacco and alcohol	4	1	1	1,768,986	0.61	0.8	336,107	Population

Breast Cancer	Treat early stage breast cancer with appropriate multimodal approaches, including generic chemotherapy, with curative intent, for cases that are referred from health centers and first-level hospitals following detection using clinical examination	4	4	1	1,496,070	0.1	0.8	1,047,249	Referral Hospital

Cervical Cancer	Opportunistic screening for cervical cancer using visual inspection or HPV DNA testing and treatment of precancerous lesions with cryotherapy	3	3	1	8,295,680	0.14	0.6	3,816,013	Health Center

Cervical Cancer	School-based HPV vaccination for girls	3	3	1	10,835,837	0.02	0.8	8,451,953	Community

Cervical Cancer	Early detection and treatment of early-stage cervical cancer	0	4	1	380,650	0.6	0.8	114,195	First-Level Hospital

Cancer-Lymphoma/ leukemia	Treatment of early-stage childhood cancers (such as Burkitt and Hodgkin lymphoma, acute lymphoblastic leukemia, retinoblastoma, and Wilms tumor) with curative intent in pediatric cancer units or hospitals	3	5	1	205,957	0.1	0.5	82,383	Referral Hospital

Cardiovascular	Long term management of ischemic heart disease, stroke, and peripheral vascular disease with aspirin, beta blockers, ACEi, and statins (as indicated) to reduce risk of further events	2	2	1	109,888,784	0.8	1.0	32,966,635	Health Center

Cardiovascular	Mass media messages concerning healthy eating or physical activity	4	1	1	1,768,986	0.23	0.8	1,008,322	Population

Cardiovascular	Opportunistic screening for hypertension for all adults and initiation of treatment among individuals with severe hypertension and/or multiple risk factors	1	1	1	20,334,580	0.442	0.8	7,279,780	Health Center

Cardiovascular	Screening and management of hypertensive disorders in pregnancy	1	3	3	452,107	0.6	0.8	180,843	Health Center

Cardiovascular	Provision of aspirin for all cases of suspected acute myocardial infarction	4	2	1	4,330	0.1	0.5	1,732	Health Center


## DISCUSSION

The findings of this Commission suggest that NCDIs comprise a large share of the burden of disease in Kenya and affect the population at younger ages than commonly believed. The burden of NCDI conditions is very diverse, and the majority of NCD DALYs in Kenya are due to conditions other than the four emphasized in global monitoring frameworks. While behavioral risk factors have a large attributable risk for NCDs, the findings of this Commission show that most of the NCDI disease burden in Kenya cannot be directly attributed to individual lifestyle choices using existing data sources. Data from HDSS sites from both rural and urban contexts demonstrate an equal, if not higher, proportion of deaths due to NCDIs in the poorest as compared to wealthiest quintiles. Furthermore, crude death rates among the poor were more than double for NCDs and triple for injuries than that among the wealthier populations.

The Kenyan National Strategy for the Prevention and Control of NCDs 2015–2020 went beyond the WHO GAP framework in including additional conditions, such as violence and injuries, palliative care, mental disorders, cognitive impairment, renal disorders, hepatic disorders, endocrine disorders, neurological conditions, hemoglobinopathies, gastroenterological, musculoskeletal, skin disorders, oral diseases, disabilities including visual and hearing impairment, and genetic disorders [35]. The Kenya Essential Package for Health also followed this model by developing a comprehensive package of services for NCDIs at multiple tiers of the health system [36]. However, this Commission found that availability of key medications and readiness of NCD services remains limited and inversely related to the poverty level of regions. Coverage of basic NCDIs, such as diagnosis and treatment of hypertension and diabetes or cancer screening, is low, and is inversely related to wealth. Domestic financing for NCDIs was also limited and not commensurate to the burden.

The interventions selected and prioritized by this Commission will require design, implementation, integration, and scale of a complex set of health sector interventions, some of which already exist within the health care system, and others that have yet to be introduced. Although this package of interventions for NCDIs is quite comprehensive, including surgical, mental health, rehabilitation, and palliative care services, it would represent only a 15.0% increase in total health expenditure or $11.76 per capita annually. This level of additional expenditure for NCDIs may not be unreasonable in the setting of recent recommendations for government expenditure on health care, such as 5% of GDP or a per capita expenditure of $86 USD in low-income countries [37].

These interventions, if implemented to target coverage, are projected to avert 9,322 premature deaths per year by the year 2030. This figure represents an approximate 10% reduction in expected premature deaths in the year 2030 (according to 2015 death rates). However, although this figure provides a reasonable estimate of averted deaths, given the greater number of interventions selected by the Kenya NCDI Poverty Commission than the DCP3 high-priority essential package, this figure is likely underestimated. Furthermore, this analysis does not include averted morbidity, which would be considerably greater than averted mortality, and provide substantial benefit to many more individuals, particularly given the emphasis on interventions for severe conditions affecting those at younger ages.

The majority of interventions selected for introduction or scale up would be applied at the primary care or health center level. Essential components to the introduction and scale-up of these interventions are many, and would include infrastructure, staffing, training, guidelines, medications, equipment, diagnostics, and referral networks. However, most of these interventions exist at some baseline level in public health sector facilities, though availability and readiness may vary dramatically. Integration with existing infrastructure and personnel for other chronic diseases, such as antiretroviral therapy for HIV/AIDS, maternal and child services and surgical services may facilitate integration of other chronic disease services for chronic NCDs and mental health conditions [38].

Health system interventions alone are not sufficient to prevent the risk and impact of NCDIs as well as provide care for NCDs on the Kenyan community, and a truly comprehensive response to NCDIs in Kenya requires a whole-of-government approach. In conjunction with the findings of this Commission, a national NCD inter-sectoral coordinating mechanism was launched to foster inter-sectoral interventions and to harness the support and synergies from outside of the health sector. This inter-sectoral committee adopted the recommendations of this Commission and is working towards broadening Kenya’s attention beyond the traditional NCDI behavioral risk factors and a more comprehensive approach rooted in equity. The recommended interventions require local adaptation and integration into existing health services platforms, coupled with development and strengthening of human resource capacity, supply chains, and referral pathways.

Mitigating the impoverishing effects of NCDIs will require the expanded coverage of the national social insurance program, establishment and strengthening of social safety nets for the poor, and increased focus on more equitable access to both preventive and curative health services through coherent cross-sectoral policies and plans. There is need to expand investment in the NCDI response via increases in direct domestic financing through capitation, insurance revenues, and innovative financing mechanisms and partnerships. With these recommendations, this Commission believes that an expansive reframing of NCDIs will allow for a comprehensive and equitable response to NCDIs in hopes of realization of the broader goal of UHC for the Kenyan population.

## References

[1] World Health Organization. Global action plan for the prevention and control of noncommunicable diseases 2013–2020 World Health Organization; 2013.

[2] Achoki T, Miller-Petrie MK, Glenn SD, et al. Health disparities across the counties of Kenya and implications for policy makers, 1990–2016: A systematic analysis for the Global Burden of Disease Study 2016. The Lancet Global Health. 1 1, 2019; 7(1): e81–95. DOI: 10.1016/S2214-109X(18)30472-8 30482677 PMC6293072

[3] Bukhman G, Mocumbi AO, Horton R. Reframing NCDs and injuries for the poorest billion: A Lancet Commission. Lancet. 2015; 386(10000): 1221–2. DOI: 10.1016/S0140-6736(15)00278-0 26403914

[4] Hulzebosch A, van de Vijver S, Oti SO, Egondi T, Kyobutungi C. Profile of people with hypertension in Nairobi’s slums: A descriptive study. Globalization and Health. 12 1, 2015; 11(1): 26 DOI: 10.1186/s12992-015-0112-1 26116577 PMC4491223

[5] Ayah R, Joshi MD, Wanjiru R, et al. A population-based survey of prevalence of diabetes and correlates in an urban slum community in Nairobi, Kenya. BMC Public Health. 12 2013; 13(1): 1–1. DOI: 10.1186/1471-2458-13-371 23601475 PMC3641964

[6] Mathenge W, Foster A, Kuper H. Urbanization, ethnicity, and cardiovascular risk in a population in transition in Nakuru, Kenya: A population-based survey. BMC Public Health. 12 1, 2010; 10(1): 569 DOI: 10.1186/1471-2458-10-569 20860807 PMC2956724

[7] Ploubidis GB, Mathenge W, De Stavola B, Grundy E, Foster A, Kuper H. Socioeconomic position and later life prevalence of hypertension, diabetes, and visual impairment in Nakuru, Kenya. International Journal of Public Health. 2 1, 2013; 58(1): 133–41. DOI: 10.1007/s00038-012-0389-2 22814479

[8] Ait-Khaled N, Odhiambo J, Pearce N, et al. Prevalence of symptoms of asthma, rhinitis, and eczema in 13-to-14-year-old children in Africa: The International Study of Asthma and Allergies in Childhood Phase III. Allergy. 3 2007; 62(3): 247–58. DOI: 10.1111/j.1398-9995.2007.01325.x 17298341

[9] Esamai F, Ayaya S, Nyandiko W. Prevalence of asthma, allergic rhinitis, and dermatitis in primary school children in Uasin Gishu district, Kenya. East African Medical Journal. 2002; 79(10): 514–8. DOI: 10.4314/eamj.v79i10.8812 12635755

[10] Patel K, Wakhisi J, Mining S, Mwangi A, Patel R. Esophageal cancer, the topmost cancer at MTRH in the Rift Valley, Kenya, and its potential risk factors. International Scholarly Research Notices. 2013 DOI: 10.1155/2013/503249 PMC3893746 24490085

[11] Mbuba CK, Ngugi AK, Fegan G, et al. Risk factors associated with the epilepsy treatment gap in Kilifi, Kenya: A cross-sectional study. The Lancet Neurology. 8 1, 2012; 11(8): 688–96. DOI: 10.1016/S1474-4422(12)70155-2 22770914 PMC3404220

[12] Jenkins R, Othieno C, Ongeri L, et al. Common mental disorder in Nyanza province, Kenya in 2013 and its associated risk factors–an assessment of change since 2004, using a repeat household survey in a demographic surveillance site. BMC Psychiatry. 12 1, 2015; 15(1): 309 DOI: 10.1186/s12888-015-0693-5 26651332 PMC4673710

[13] Jenkins R, Othieno C, Omollo R, et al. Tedium vitae, death wishes, suicidal ideation, and attempts in Kenya-prevalence and risk factors. BMC Public Health. 12 1, 2015; 15(1): 759 DOI: 10.1186/s12889-015-2089-3 26253319 PMC4528694

[14] Kenya Ministry of Health. Kenya Stepwise Survey for Noncommunicable Diseases Risk Factors 2015 Report Division of Noncommunicable Diseases, Kenya Ministry of Health, 2015.

[15] Africa Population Health Research Center. Nairobi Urban Health Demographic Surveillance System Indicators 2003–2015. Nairobi: African Population and Health Research Center; 2017.

[16] Bamiwuye SO, Odimegwu C. Spousal violence in sub-Saharan Africa: Does household poverty-wealth matter? Reproductive Health. 12 1, 2014; 11(1): 45 DOI: 10.1186/1742-4755-11-45 24935486 PMC4076508

[17] Lawoko S, Dalal K, Jiayou L, Jansson B. Social inequalities in intimate partner violence: A study of women in Kenya. Violence and Victims. 12 1, 2007; 22(6): 773–84. DOI: 10.1891/088667007782793101 18225388

[18] Kenya National Bureau of Statistics, Ministry of Health, National AIDS Control Council, et al. Kenya Demographic and Health Survey 2014. 2015 https://www.dhsprogram.com/pubs/pdf/FR308/FR308.pdf. Accessed September 20, 2018.

[19] Ombati AN, Ndaguatha PL, Wanjeri JK. Risk factors for kerosene stove explosion burns seen at Kenyatta National Hospital in Kenya. Burns. 5 1, 2013; 39(3): 501–6. DOI: 10.1016/j.burns.2012.07.008 22999210

[20] Mwai D, Muriithi M. Economic effects of non-communicable diseases on household income in Kenya: A comparative analysis perspective. Public Health Res. 2016; 6(3): 83–90.

[21] Mwai D, Muriithi M. Catastrophic health expenditure and household impoverishment: A case of NCDs prevalence in Kenya. Epidemiology, Biostatistics, and Public Health. 3 21, 2016; 13(1).

[22] Kenya Ministry of Health. The Kenya Non-Communicable Diseases & Injuries Poverty Commission Report Kenya Ministry of Health; 2018 http://www.ncdipoverty.org/kenya-report.

[23] Institute for Health Metrics and Evaluation (IHME). GBD Compare Seattle, WA: IHME, University of Washington; 2015 http://vizhub.healthdata.org/gbd-compare. Accessed December 4, 2017.

[24] Kenya Medical Research Institute (KEMRI) & Centers for Disease Control (CDC) Health Demographic Surveillance Site (HDSS). Data provided courtesy of KEMRI Director. 2017.

[25] Alkire S, Robles G. Global Multidimensional Poverty Index 2017. OPHI Briefing 47, 2017.

[26] Government of Kenya. Kenya Service Availability and Readiness Assessment Mapping (SARAM). Kenya Ministry of Health; 2014.

[27] Kenya Ministry of Health. Kenya Essential Medicines List 2016 Kenya Ministry of Health; 2016.

[28] Kenya Ministry of Health. Kenya National Health Accounts 2015/2016 Kenya Ministry of Health; 2017.

[29] Korir A, Okerosi N, Ronoh V, Mutuma G, Parkin M. Incidence of cancer in Nairobi, Kenya (2004–2008). International Journal of Cancer. 11 1, 2015; 137(9): 2053–9. DOI: 10.1002/ijc.29674 26139540

[30] Ali Z. Kenya Hospices and Palliative Care Association: Integrating palliative care in public hospitals in Kenya. Ecancermedicalscience. 2016; 10 DOI: 10.3332/ecancer.2016.655 PMC4970621 27563350

[31] World Health Organization. Making fair choices on the path to universal health coverage: Final report of the WHO Consultative Group on Equity and Universal Health Coverage World Health Organization; 2014. 10.2471/BLT.14.139139 PMC4047814 24940009

[32] Jamison DT, Alwan A, Mock CN, et al. Universal health coverage and intersectoral action for health: key messages from Disease Control Priorities. The Lancet. 3 17; 2018; 391(10125): 1108–20. DOI: 10.1016/S0140-6736(15)60097-6 PMC5996988 29179954

[33] Watkins D, Qi J, Horton S. Costs and affordability of essential universal health coverage in low-and middle-income countries. Disease Control Priorities. 2017 http://dcp-3.org/resources/costs-and-affordability-essential-universal-health-coverage-low-and-middle-income.

[34] Watkins DA, Norheim OF, Jha P, Jamison DT. Mortality Impact of Acheiving Essential Universal Health Coverage in Low-and Lower Middle-Income Countries. Disease Control Priorities. 2017 http://dcp-3.org/resources/mortality-impact-achieving-essential-universal-health-coverage-low-and-middle-income.

[35] Kenya Ministry of Health. Kenyan National Strategy for the Prevention and Control of NCDs 2015–2020 Kenya Ministry of Health; 2015.

[36] Kenya Ministry of Medical Services and Kenya Ministry of Public Health & Sanitation. Accelerating Attainment of Health Goals: The Kenya Health Sector Strategic and Investment Plan: July 2013–June 2017 Kenya Ministry of Medical Services and Ministry of Public Health & Sanitation; 2012.

[37] Meheus F, McIntyre D. Fiscal space for domestic funding of health and other social services. Health Economics, Policy and Law. 4 2017; 12(2): 159–77. DOI: 10.1017/S1744133116000438 28332459

[38] Gupta N, Bukhman G. Leveraging the lessons learned from HIV/AIDS for coordinated chronic care delivery in resource-poor settings. Healthcare. 2015; 3(4): 215–20. DOI: 10.1016/j.hjdsi.2015.09.006 26699346

